# Human head–neck model and its application thresholds: a narrative review

**DOI:** 10.1097/JS9.0000000000001941

**Published:** 2024-07-11

**Authors:** Ziyang Liang, Ke Wu, Tengfei Tian, Fuhao Mo

**Affiliations:** a State Key Laboratory of Advanced Design and Manufacturing Technology for Vehicle, Hunan University; b Xiangjiang Laboratory, Changsha, Hunan; c Department of Tuina and Spinal Orthopedics in Chinese Medicine, Shenzhen Traditional Chinese Medicine Hospital, The Fourth Clinical Medical College of Guangzhou University of Chinese Medicine, Shenzhen, Guangdong, People’s Republic of China

**Keywords:** application, finite element method, human head–neck, multibody method, validation

## Abstract

There have been many studies on human head–neck biomechanical models in the last two decades, and the associated modelling techniques were constantly evolving at the same time. Computational approaches have been widely leveraged, in parallel to conventional physical tests, to investigate biomechanics and injuries of the head–neck system in fields like the automotive industry, orthopedic, sports medicine, etc. The purpose of this manuscript is to provide a global review of the existing knowledge related to the modelling approaches, structural and biomechanical characteristics, validation, and application of the present head–neck models. This endeavor aims to support further enhancements and validations in modelling practices, particularly addressing the lack of data for model validation, as well as to prospect future advances in terms of the topics. Seventy-four models subject to the proposed selection criteria are considered. Based on previously established and validated head–neck computational models, most of the studies performed in-depth investigations of included cases, which revolved around four specific subjects: physiopathology, treatment evaluation, collision condition, and sports injury. Through the review of the recent 20 years of research, the summarized modelling information indicated existing deficiencies and future research topics, as well as provided references for subsequent head–neck model development and application.

## Introduction

Highlights

- Seventy-four models were performed in in-depth investigations during the last two decades, and methodologies primarily focused on application-centered differences, which revolved around four specific subjects: physiopathology, treatment evaluation, collision condition, and sports injury.
- Advanced neuromuscular reflex control is being included in biomechanical head–neck models for collision applications, while this important control strategy has yet to be added to the medical models.
- Standardization in integrated medicine and engineering can greatly enhance the development and application of modelling processes in this field. By establishing standardized practices, guidelines, and frameworks, the integration of medicine and engineering can become more efficient, reliable, and effective.

Pathogenesis, prevention, treatment, and rehabilitation efforts became the main research directions of head–neck injury biomechanics. To study cervical spine injuries in trauma events in detail, numerical simulation plays an important role in those efforts. It is thus not a surprise that the biomechanical model of the cervical spine has expanded at a rapid pace. Based on the early technique of frequent computational modelling for kinematic analysis^1–5^, human body models (HBMs) at varying levels have been created by various researchers globally in the last two decades. Previous finite element (FE) or multibody (MB) methods were established and contributed to a better understanding of neck injury mechanisms, which could be used in injury prevention design. Examples included whiplash injuries in traffic accidents, mechanical neck pain in occupational health issues, and degenerative disc diseases in medical intervention. At the application level, several studies developed protective helmets based on the aforementioned models to minimize head and neck injuries under impact loads^6,7^. Additionally, research has been conducted on the development of cervical traction exoskeletons to aid in the rehabilitation of muscular and neurological injuries in the head and neck region^8–11^.

However, those models often cast doubt on credibility due to their weak universality^12,13^. In addition, the MB was employed to analyze the dynamics and motion of the cervical spine or head–neck region, but it cannot easily and accurately obtain tissue stress and strain distribution for detailed injury risk analysis, which probably limits its application. At the beginning of this century, several whole HBMs were developed for injury analysis with the rise of computational resources becoming more accessible^14–17^. These models are designed to estimate injury under collision conditions regarding automotive safety but are used less in other environments. The development of imaging technologies, in particular, the improved accuracy of computed tomography (CT), makes it possible to reconstruct individualized three-dimensional geometry. Researchers tend to establish individualized models to evaluate and monitor medical interventions in medical simulations^18,19^. These scenarios include but are not limited to the assessment of driving comfort and the evaluation of motion sickness^20–22^. In the last two decades, HBMs have had an increasing number of application scenarios. It is believed that models of various applications exhibit disciplinary differences, such as modelling approaches, material properties, validations, etc.

**Figure 1:**
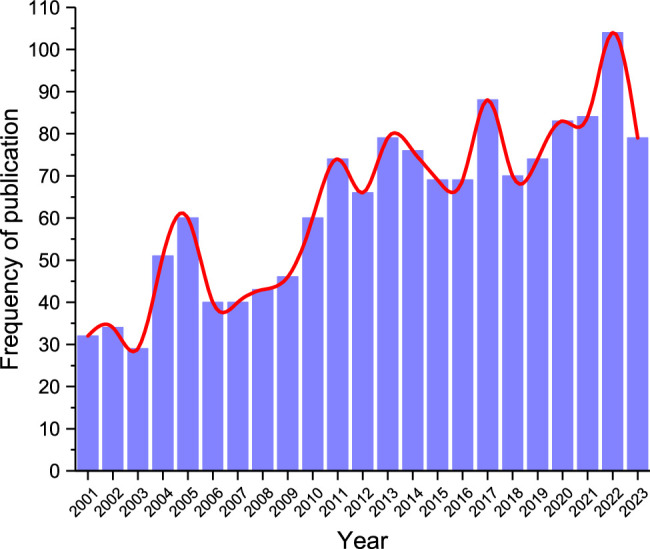
Frequency of published studies by year.

This study aimed to provide a global review of the present knowledge related to human head–neck models over the past two decades. The analysis was performed from global knowledge to dedicated focus on the biomechanical modelling researchers targeted in this work. As shown in Figure 1, the PubMed search terms ‘cervical spine’ and ‘biomechanics model’ showed that the number of articles in this field has increased exponentially over the period^23^. In addition, this review is intended to provide a thorough understanding of the current state-of-the-art techniques and help inform future developmental efforts to create more accurate and realistic cervical spine models. Although there is a wide array of computational cervical spine models, they have yet to be compared to one another in various aspects of multidisciplinary thresholds to provide information that could be used to improve them so that they better serve current needs. Thus, the modelling approach, structural characteristics, validation, and application of existing computer head–neck models were compared and analyzed. Furthermore, this review outlines what researchers from different backgrounds have achieved with cervical spine models and describes the synthesis of characteristics from multiple disciplines, clarifying the gap in biomechanical head–neck models of interdisciplinary research.

## Methods

**Figure 2:**
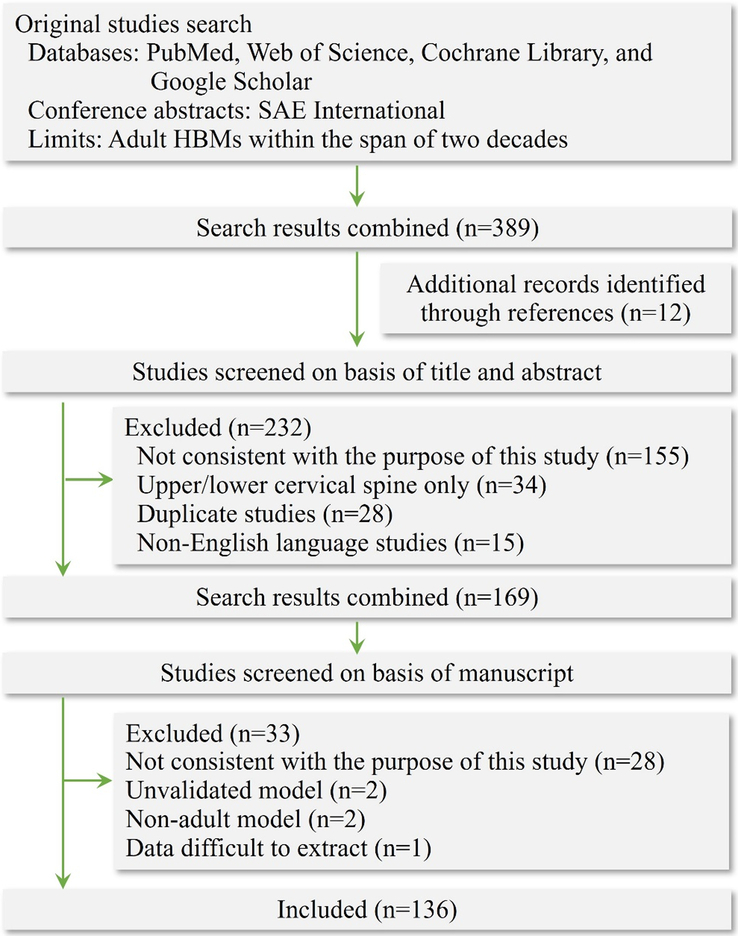
Flowchart illustrating literature search and selection process.

Research groups working in the field of cervical spine FE modelling were included in the present study. Only validated models of the head–neck model that were previously published in peer-reviewed journals were considered. This study included common simulation methods that employ either MB models or FE models. The modelling approach, structural characteristics, and validation of different models were reviewed in detail. The selection criteria for studies included in the search were ‘Cervical spine’ OR ‘Head-neck,’ ‘Multibody model’ OR ‘Finite element model,’ Adult HBMs within two decades, Publication in the ‘English’ language. The exclusion criteria were only investigated the upper cervical spine (C0–C2) as well as the lower cervical spine (C3–C7) and unvalidated models, which are regarded as imprecise. The Boolean operators ‘AND,’ ‘OR,’ ‘NOT,’ and the proximity operators ‘PRE/n’ were used to search relevant studies, which were performed through PubMed, Web of Science, Cochrane Library, SAE International, and Google Scholar. Articles cited in all retrieved studies were also searched to gather additional sources, including the grey literature. There were three researchers conducted this survey and collectively reviewed studies that were on the border of inclusion/exclusion criteria. Components evaluated include anatomical structure modelling, muscle structure and its status (active/passive/none), muscle activation type (predefined/feedback-based optimization/EMG driven mode/neuromuscular feedback), personalized muscle parameters, and validation methods (dynamic/quasi-static). A flowchart illustrating the study search and selection process is shown in Figure 2.

## Results

### Summary of modelling, validation, and application

A total of 74 neck or hea–-neck models satisfy our selection criteria in this review. Fifty-nine FE models and 15 MB models are reported. Tables 1 and 2 briefly illustrate the modelling approach, structural characteristics, validations, and applications of these models, respectively. The models are chronologically updated in terms of almost all relevant aspects, for example, from simple to complicated in geometry and anatomy, from rigid-body to deformable on tissue material property, and from passive to active on muscle effect. Advances in imaging technologies, for example, CT and MRI, as well as in controlling theory, are essentially beneficial in this evolution. Significant progress has been made in the study of modelling materials, techniques, and valuation methods during the last two decades^24^. Recent numerical models also tried to include more complicated control strategies, from simple proportional-integral-derivative (PID) control to complex physiological neural feedback control strategies.

**Table 1:** Multipage tables of human head–neck finite element models within retrieved studies in the last two decades.

First author/year	Robin, 2001 (HUMOS)^43^	Ng, 2004^67^	Meyer, 2004^44^	Brolin, 2005 (KTH)^36^	Ejima, 2005 (JAMA)^45^
Type	FE	FE	FE	FE	FE
Software	Radioss	ANSYS	Radioss	LS-DYNA	LS-DYNA
Segments	Whole body model	C2–C7	Head–T1	Head–T1	Whole body model
Figure	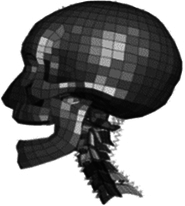	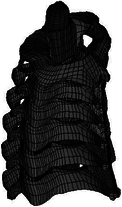	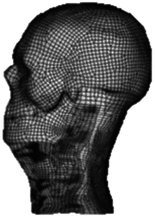	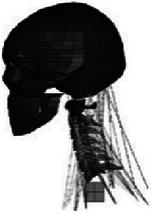	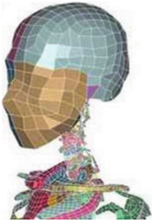
Geometry	CT	3D digitizer	CT	CT	MRI
Head	Viscoelastic	N/A	Rigid/elastoplastic orthotropic^68^	Rigid	Head-T1
Vertebra	Elastoplastic	Elastic	Rigid	Viscoelastic/rigid	N/A
Disc	Elastic	Elastic	Elastic	Elastic, anisotropic (annulus fibrosis); visco-elastic (annulus matrix); elastic, incompressible (nucleus pulposus)	N/A
Facet joint	Contact	Contact	Contact	Contact	Share nodes
Ligament	Elastic	Nonlinear cable	Nonlinear spring	Nonlinear beam	N/A
Spinal canal	N/A	N/A	Elastic (cerebrospinal fluid)^68^	Elastic (dura, pia) incompressible fluid (cerebrospinal fluid)^69^	N/A
Muscle	Solid with nonlinear springs (passive muscle)	N/A	1D Hill-type beam (active muscle)^68^ Solid element (passive muscle)	1D Hill-type beam (active muscle) 3D solid (passive muscle)^70,71^	3D solid (passive muscle)
Muscle activation	N/A	N/A	Predefined^68^	Predefined/EMG^71^	N/A
Muscle structure	N/A	N/A	N/A	Cadaver measurement/MRI modelling^70,71^	MRI modelling
Elements	9223 solids 13 458 shells 504 springs	18 731 elements	9223 solids 13 458 shells 504 springs	N/A	14 734 nodes 7306 solids 6976 shells
Validation	15 g frontal impact 7 g lateral impact 10 g oblique impact^72^	Quasi-static Flx/Ext/LB/AR^73,74^	15 g frontal impact/7 g lateral impact^75,76^; oblique impact^77^; rear impact^78^	Quasi-static compression/shear/ torsion for LCS^5,79,80^; Flx/Ext/LB/AR/tension for UCS^81–84^; compression/Flx/LB/oblique impacts for whole cervical spine^5,72,85–89^; frontal/rear/lateral/oblique impacts^72,87,90^	4 g rear impact^91^
Application	In Tropiano, 2004^92^: whiplash trauma simulation based on the HUMOS model^43^ by imposing a rear impact	In Ng, 2004, 2005^39,67,93^: intersegment stability and osteophyte formation in C2–C7 laminectomy with unilateral and bilateral facetectomy	In Meyer, 2013, 2019, 2021^68,94,95^: injury risk criteria and forces/moments in cervical segments based on Meyer, 2004^44^ under different impact scenarios	In Kleiven, 2006^69^, Hedenstierna, 2008, 2009^70,71^: Evaluation of head injury criteria and neck muscles responses based on the KTH model^36^ during different impact scenarios	Evaluation of neck passive muscles responses during rear impact
First author/year	Zhang, 2006^40^	Frechede, 2006^96^	del Palomar, 2008^41^	Kitagawa, 2008 (THUMS)^15^	Tchako, 2009^42^
Type	FE	FE	FE	FE	FE
Software	ANSYS/LS-DYNA	RADIOSS	ABAQUS	LS-DYNA	ANSYS
Segments	Head–C7	Head–T1	C1–C7	Whole body model	C1–T1
Figure	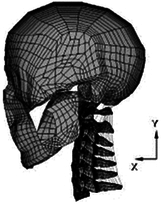	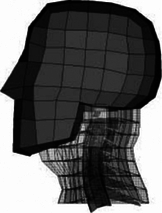	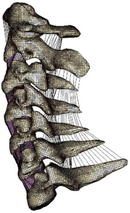	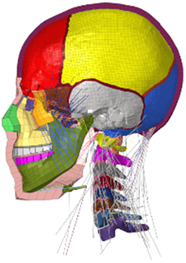	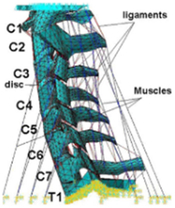
Geometry	3D digitizer	Hybrid III dummy (Head) Geometric modelling (C2-T1)	CT	CT	Digitized geometrical measurement
Head	Elastic	Rigid	N/A	Hyperelastic	N/A
Vertebra	Elastic	Rigid	Rigid	Elasto-viscoplastic (cortical bone) Elastic viscoplastic (cancellous bone)	Elastic
Disc	Elastic	Viscoelastic	Hyperelastic (annulus matrix/nucleus pulposus); elastic (annulus fibrosis)	Seatbelt (annulus fibrosis); viscoelastic (annulus matrix); isotropic plasticity (nucleus pulposus)	Elastic
Facet joint	Contact	Contact	Frictional contact	Contact	Contact
Ligament	Elastic/nonlinear link^97^	Viscoelastic spring	Nonlinear truss	Nonlinear shell	Nonlinear spring
Spinal canal	N/A	N/A	N/A	Hyperelastic (epidural fat) Orthotropic nonlinear elastic (dura, pia) Incompressible fluid (cerebrospinal fluid) Viscoelastic (cord)	N/A
Muscle	1D springs (passive muscle)^98^	3D viscoelastic solid (passive muscle)	N/A	Hill-type 1D beam (active muscle)^99^ 3D hyperelastic solid (passive muscle)	1D elastic spring (passive muscle)
Muscle activation	N/A	N/A	N/A	EMG^99,100^	N/A
Muscle structure	Cadaver measurement	Cadaver measurement	N/A	Visible human subject^101^	Cadaver measurement
Elements	22 094 elements	N/A	N/A	About 80 000 elements	19 953 solids 7605 shells 434 springs/cables
Validation	Quasi-static Flx/Ext/LB/AR^73,74,102–107^; 5 g/8 g rear impact^108^; In Zhang *et al*.^109^: 3.2 m/s drop test^110^; 2.6 m/s rear impact^111^	15 g frontal impact 7 g lateral impact 10 g oblique impact 7 g rear impact^72,78,112–114^	Quasi-static Flx/Ext/LB/AR^40,102,115^	3 g/15 g frontal impact^75,116–118^ 7 g lateral impact^119^ 4 g rear impact^91^ Dynamic axial loading^86^	Quasi-static Flx/Ext/LB/AR^74,120,121^
Application	In Teo, 2007^122^, Zhang, 2008^97,98^: Head–neck kinematics based on Zhang, 2004^40^ during different impact orientations/accelerations	Relation between curvature and risk of injury during omni-directional impacts	Mechanisms of disc injury (maximum shear strains) under quasi-static loading	In Iwamoto, 2009^99^, Kato, 2018^101^: Muscle Effects of muscle activity in pre-impact on the bone responses based on the THUMS model^15^	In Tchako, 2009^42,123^: external and internal responses of the spinal components to disc herniation and clinical instability under diving and football accidents; simulated stress response in intervertebral discs of partial discectomies and fusion surgeries
First author/year	Laville, 2009^55^	Espinha, 2010^124^	Kallemeyn, 2010^125^	Li, 2010^126^	Toosizadeh, 2011^127^
Type	FE	FE	FE	FE	FE
Software	Radioss	ABAQUS	ABAQUS	ABAQUS	ANSYS
Segments	C0–T4	C1–T1	C2–C7	C1–C7	C0–C7
Figure	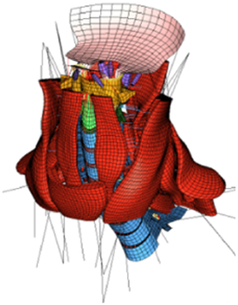	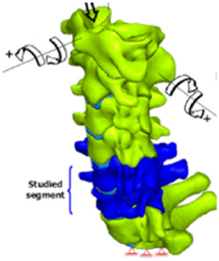	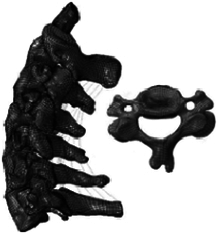	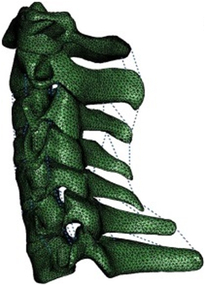	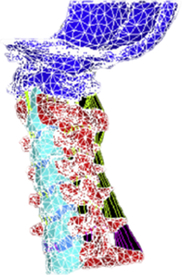
Geometry	EOS/MRI (biplanar X-rays)	CT	CT	CT	CT
Head	Rigid	N/A	N/A	N/A	Rigid
Vertebra	Elastic	Elastic	Elastic	Anisotropy elastic (cortical/cancellous bone)	Orthotropic/isotropic elastic
Disc	Elastic	Elastic	Elastic (annulus fibrosis/matrix) Incompressible fluid (nucleus pulposus)	Elastic	Hyperelastic (annulus matrix) Nonlinear link (annulus fibrosis) Incompressible fluid (nucleus pulposus)
Facet joint	Contact	Hard contact	Elastic	N/A	Frictional contact
Ligament	Bilinear spring/shell	N/A	Nonlinear truss	Elastic	Nonlinear spring
Spinal canal	N/A	N/A	N/A	N/A	N/A
Muscle	Hill-type 1D spring (active muscle) 3D solid (passive muscle)	N/A	N/A	N/A	Optimization (equilibrium algorithm)
Muscle activation	Predefined	N/A	N/A	N/A	N/A
Muscle structure	MRI modelling	N/A	N/A	N/A	N/A
Elements	37 000 solids 30 500 shells 41 500 springs 60 000 nodes	N/A	About 130 000 elements	264 301 elements 67 033 nodes	N/A
Validation	Quasi-static compression/Flx/Ext/LB/AR^73,74,115,128–131^	Physiological model measurement for bone mass	Quasi-static Flx/Ext/LB/AR (in house experiments)	Quasi-static Flx/Ext/LB/AR^40,73,109,115^	Quasi-static Flx/Ext/LB/AR^40,104,115,132^
Application	Influence of geometrical parameters (cervical spine) on the motion patterns	Influence of the spine instrumentation on bone remodelling (fusion process) after an ACDF	In Kode, 2014^133^, Gandhi, 2019^134^, Stoner, 2020^135^: Based on Kallemeyn, 2010^125^, investigated kinematics and stress/strain responses (tissue/instrument) of disc degeneration, multi-level laminoplasty, laminectomy, ACDF, and ADR	In Li, 2010^126^: Association between degenerative disc extent and responses of cervical spine; In Qi, 2016^136^: Kinematics and stress (instrument/graft material) response of ACD, ACDF, and ADR	Relationship between required muscle forces and neck angle for the quasi-static condition (Risk factor for neck pain and arthritis)
First author/year	Panzer, 2011^137^	Zhang, 2011^138^	Lee, 2011^18^	Rosli, 2011^139^	Wagnac, 2012 (S2MS)^140^
Type	FE	FE	FE	FE	FE
Software	LS-DYNA	LS-DYNA	ABAQUS	CosmosWork	Radioss
Segments	Head–T1	C1–T1	C2–C7	C1–C7	Complete spine
Figure	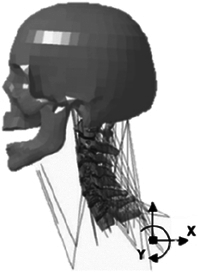	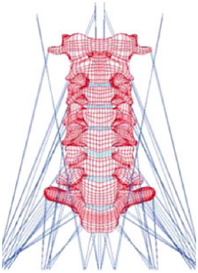	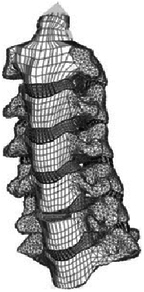	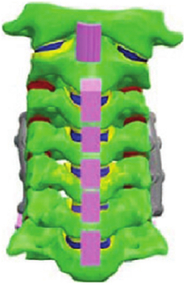	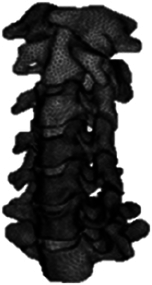
Geometry	CT	CT	CT	CT	CT
Head	Rigid	Rigid	N/A	N/A	N/A
Vertebra	Rigid	Elastic	Elastic	Elastic	Elastoplastic
Disc	Hyperelastic (annulus matrix) Nonlinear elastic (annulus fibrosis) Linear visoelastic (nucleus pulposus)	Elastic	Elastic	N/A	Viscoelastic
Facet joint	Contact	N/A	Contact	N/A	Contact
Ligament	Nonlinear spring	Elastic cable/solid	Elastic	Elastic	Nonlinear spring/shell
Spinal canal	N/A	N/A	N/A	N/A	N/A
Muscle	Hill-type 1D beam (active muscle)	1D beam (passive muscle)	N/A	N/A	N/A
Muscle activation	Predefined	N/A	N/A	N/A	N/A
Muscle structure	Cadaver measurement	Literatures observations	N/A	N/A	N/A
Elements	108 354 elements	35 042 nodes 22 618 elements	38 984 nodes 77 991 elements	N/A	740 000 elements
Validation	8 g/22 g frontal impact^141,142^ In Fice, 2011^54^: 4 g/7 g/8 g rear impact^143–147^ Ligaments strain threshold^148^	4 g rear impact^143^ Dynamic response in injury position^149^	Quasi-static Flx/Ext/LB/AR^67,73,150–152^	C4-6 1.0 N-m extension^73,153,154^	Quasi-static compression/Flx/Ext; dynamic Flx and Ext
Applicatiioonn	In Fice, 2012^155^, Cronin, 2014^156^, Shateri, 2015^157^: Biofidelic kinematic and local tissue response of full head-neck in different impact scenarios based on Panzer and Fice’s model^54,137^	Kinematic and local tissue response of cervical spine in whiplash injury	In Lee, 2016^158^: biomechanical response of different types in ADR devices based on the Lee, 2011^18^	In Rosli, 2014^159^: stability of cervical spine after one-level corpectomy using different numbers of screws and plate systems based on the Rosli, 2011^139^	Healthy spine subject approximating to the 50th percentile of European male
First author/year	Gayzik, 2012 (GHBMC)^37^	Wei, 2013^160^	Xie, 2013^161^	Mesfar, 2013^162^	Erbulut, 2014^163^
Type	FE	FE	FE	FE	FE
Software	LS-DYNA	ANSYS	ABAQUS	ABAQUS	ABAQUS
Segments	Whole body model	C1–C7	C2–C7	Head–T1	C2–T1
Figure	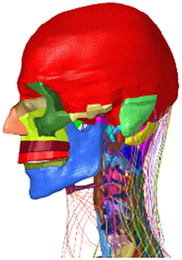	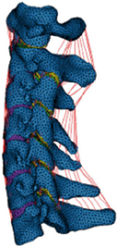	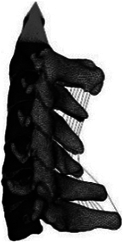	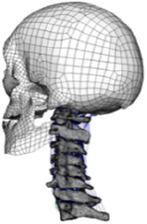	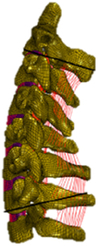
Geometry	MRI and CT	CT	CT	CT/MRI	CT
Head	Viscoelastic	N/A	N/A	Rigid	N/A
Vertebra	Elastoplastic	Elastic	Elastic	Rigid	Elastic
Disc	Viscoelastic (annulus matrix) Orthotropic nonlinear elastic (annulus fibrosis) Elastic fluid (nucleus pulposus)	Elastic	Elastic	Elastic	Elastic
Facet joint	Contact	Contact	Frictionless contact	Contact	Contact
Ligament	Nonlinear spring	Nonlinear spring	Nonlinear spring	Nonlinear spring/shell	Elastic truss
Spinal canal	Visoelastic (spinal cord/ cerebrospinal fluid); elastic (arachnoid/dura)	N/A	N/A	N/A	N/A
Muscle	Separated muscle bundles with 3D solid elements (passive muscle) Hill-Type 1D beam (active muscle)	N/A	N/A	N/A	N/A
Muscle activation	Predefined^164^	N/A	eN/A	N/A	N/A
Muscle structure	MRI modelling	N/A	N/A	N/A	N/A
Elements	Whole body model: 2 192 031 elements	189 527 solids 62 834 shells 596 springs	352 029 elements	N/A	106 547 elements
Validation	High-rate Flx/Ext (C2–T1) [University of Waterloo experimental testing]; Quasi-static Flx/Ext (C2–T1)^115,132,149,165^; Quasi-static tension (C2–T1)^166^; Spine intervertebral junctions from C2–C3 through C6–C7 for Frontal/rear Impact, investigating strain in CL, PLL, ALL, anterior and posterior disk shear strain^141,146,147,167,168^; C0–T1 AR^169,170^; Full neck model for tension^166^; 3.6 g/7 g rear impact^77,143,171,172^; 7 g lateral impact^77,172^; 8 g/15 g front impact^77,172^	Quasi-static Flx/Ext/LB/AR^74,102^	Quasi-static Flx/Ext/LB/AR^18,40,73,115^	Quasi-static Flx-Ext^73,115^	Quasi-static Flx/Ext/LB/AR^40,115,132,137,149,173^
Application	In White, 2015^174^, Corrales, 2021^56,175,176^, Whyte, 2021^177^: base on the GHBMC model^37^, investigated neck response and kinematics under impact scenarios and multiaxial transverse shear loading; the influences of capsular joint cartilage geometry, sex, age, stature, vestibulocollic, and cervicocollic muscle reflexes under quasi-static/dynamic loading; quantifying the active muscle repositioning under flexion^178^; spinal cord affect brain tissue strains in impact^179^	Comparison between straightened and normal physiological curvature in cervical spine	Comparison among laminectomy, hemilaminectomy, and novel MIS treatment of multilevel intradural tumour	Effect of the transverse ligament rupture on biomechanical response of head-neck under a compression	In Erbulut, 2014^163^, Zafarparandeh, 2016^180^, Mumtaz, 2022^181^: role of soft tissues in stability, and sensitivity of FE model to geometry; biomechanical differences among single, multilevel and hybrid disc replacement surgery with dynamic cervical implant and fusion
First author/year	Howley, 2014^182^	Wang, 2014^183^	Chung, 2015^184^	Khuyagbaatar, 2015^19^	Wang, 2016^185^
Type	FE	FE	FE	FE	FE
Software	LS-DYNA	ABAQUS	ABAQUS	ABAQUS	ABAQUS
Segments	Head–T6	Head–T1	C2–C7	C2–C7	C2–T1
Figure	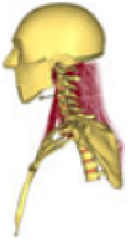	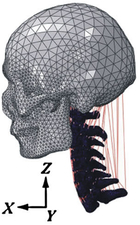	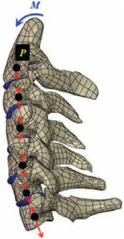	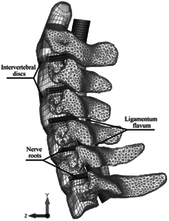	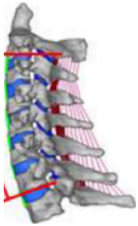
Geometry	MRI	CT	CT	CT	Visible human project/MRI
Head	Rigid	Rigid	N/A	N/A	N/A
Vertebra	Elastic	Elastic	Elastic	Rigid	Elastic
Disc	Hyperelastic (annulus matrix) Hyperelastic anisotropic transverse (annulus fibrosis) Incompressible fluid (nucleus pulposus)	Elastic	Hyperelastic (annulus fibrosis/nucleus pulposus)	Rigid	Hyperelastic
Facet joint	Contact	Frictionless contact	Frictionless contact	N/A	Frictional contact
Ligament	Bilinear spring	Elastic truss	Elastic truss	Rigid	Nonlinear spring
Spinal canal	N/A	N/A	N/A	Hyperelastic (spinal cord); elastic (dura matter/nerve roots); viscoelastic (cerebrospinal fluid)	N/A
Muscle	Hill-type 1D spring (active muscle) 3D solid (passive muscle)	N/A	N/A	N/A	N/A
Muscle activation	EMG	N/A	N/A	N/A	N/A
Muscle structure	MRI combined with personalisation (mesh deformation)	N/A	N/A	N/A	N/A
Elements	N/A	N/A	N/A	N/A	N/A
Validation	Quasi-static Flx/Ext/LB/AR	RMSE / rRMSE in quasi-static Flx/Ext/LB/AR (*in vivo* kinematic capture)	Quasi-static Flx/Ext/LB/AR^125^	Impact compression for dural sheath and whole spinal cord^186,187^	Quasi-static Flx/Ext/LB/AR^115,129,130^
Application	A framework for orthopaedic applications towards personalisation and active muscle integration in a head–neck FE model	A novel method towards in vivo experiment (motion capture) and FE model integration	Kinematics and instrument stress of two-level ADR and intervertebral Cage (hybrid cervical surgery)	In Khuyagbaatar, 2015, 2016^19,188^: Simulated different types of OPLL on mechanical stress in spinal cord, and biomechanical mechanisms in three types of SCI; in Khuyagbaatar, 2016, 2017, 2018^63,189–191^: evaluated stress and strain of spinal cord due to OPLL in the cervical spine under flexion after laminectomy; postoperative C5 palsy due to OPLL in different types of cervical spinal alignment; biomechanical effects between open-door and double-door laminoplasty on spinal cord and nerve root following laminoplasty for OPLL	In Wang, 2016, 2017^185,192^: kinematics and load-sharing pattern of resection or degeneration of uncovertebral joints altered; anterior plating failure in treating distractive flexion injury In Liu, 2021^193^: length and density of instruments in anterior/posterior cervical approach for ankylosing spondylitis cervical spine fracture
First author/year	Östh, 2016^14^	Deng, 2017^194^	Wang, 2017^195^	Rong, 2017^196^	Gadomski, 2018^197^
Type	FE	FE	FE	FE	FE
Software	LS-DYNA	ABAQUS	ABAQUS	ABAQUS	ABAQUS
Segments	Head–neck model	C2–T1	C0–T1	C2–C7	C0–C7
Figure	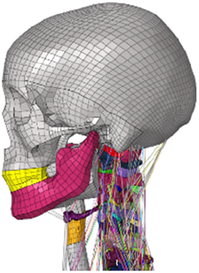	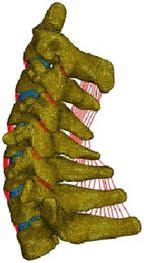	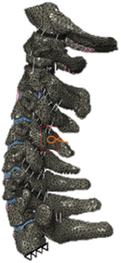	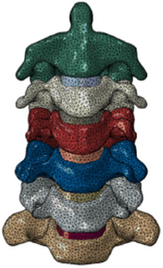	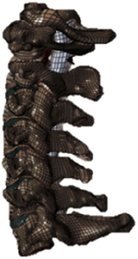
Geometry	CT	CT	CT	CT	CT
Head	Rigid	N/A	Elastic	N/A	Elastic/orthotropic
Vertebra	Elastoplastic	Elastic	Elastic	Elastic	Elastic/orthotropic
Disc	Viscoelastic (annulus matrix/nucleus pulposus) Orthotropic nonlinear elastic (annulus fibrosis)	Elastic	Elastic	Elastic	Elastic
Facet joint	Contact	Frictionless contact	Frictionless contact	Frictionless contact	Contact
Ligament	Nonlinear membrane	Elastic truss	Elastic truss	Elastic	Nonlinear spring
Spinal canal	N/A	N/A	N/A	N/A	Hyperelastic (spinal cord)
Muscle	1D beam elements of Hill muscles (active muscle)	N/A	N/A	N/A	N/A
Muscle activation	Feedback-based optimization^198^	N/A	N/A	N/A	N/A
Muscle structure	Cadaver measurement^199^	N/A	N/A	N/A	N/A
Elements	115 801 elements, 35 441 nodes	N/A	255 401 elements, 351 541 nodes	N/A	N/A
Validation	Anatomic measurements^200,201^; ligaments tension^202–204^; quasi-static Flx/Ext/LB/AR^73,115,149^; LCS segment compliance^153^; spinal alignment assessment^205^; modelling reflex recruitment of neck muscles in a HBM for simulating omnidirectional head kinematics^199^; segmental instantaneous centres of rotation evaluation^199^; 1.3 m/s and 2.6 m/s rear impact^206–208^	Quasi-static Flx/Ext/LB/AR^73,74,115,132,209^	Quasi-static Flx/Ext/LB/AR^40,73^	Quasi-static Flx/Ext/LB/AR^73,158,210^	FE-modeled motion was compared with experimentally measured motion
Application	In Putra, 2019, 2021, 2022^57,198,211^: Implemented a control strategy to neural feedback from the vestibular system and muscle spindles under whiplash injury based on the VIVA model^14,199^; active reflexive muscles suitable for sex based whiplash injury prediction^212^	Disc responses of cervical traction therapy with and without neck support; Disc nucleus pulposus pressure in cervical spine positioning rotation manipulation^213^	Disc responses of traditional Chinese manipulation	Influence of facet tropism on the intervertebral disc and facet joints in the cervical spine	Kinematics and spinal cord responses of intact cervical spines undergoing endotracheal intubation
First author/year	Mihara, 2018^214^	Yan, 2018^215^	John, 2019^216^	Park, 2019^217^	Xie, 2019^218^
Type	FE	FE	FE	FE	FE
Software	LS-DYNA	LS-DYNA	LS-DYNA	ABAQUS	ABAQUS
Segments	C2–T2	Head–neck	Head–neck	C2–C7	C2–C7
Figure	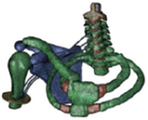	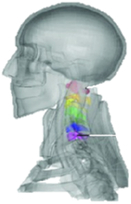	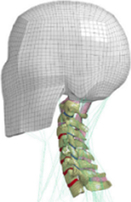	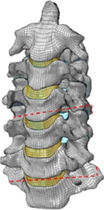	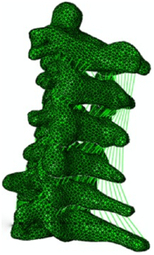
Geometry	Visible human project	MRI	CT	CT	CT
Head	N/A	Elastic	Rigid	N/A	N/A
Vertebra	Elastic	Elastoplastic	Elastoplastic	Elastic	Elastic
Disc	Elastic	Elastic	Viscoelastic (annulus matrix) Orthotropic nonlinear elastic (annulus fibrosis) Viscoelastic (nucleus pulposus)	Elastic	Elastic
Facet joint	Contact	Contact	Contact	Contact	Frictionless contact
Ligament	N/A	Nonlinear spring	Nonlinear membrane	Nonlinear truss	Nonlinear truss
Spinal canal	Elastic (dura mater/nerve root/brachial plexus)	Elastic (pia matter/falx and tentorium) Viscoelastic (cerebral/cerebellum/brain stem/cerebrospinal fluid)	N/A	N/A	N/A
Muscle	N/A	Hill-type 1D beam (active muscle) 3D solid (passive muscle)	Hill-type 1D truss (passive muscle)	N/A	N/A
Muscle activation	N/A	Predefined	N/A	N/A	N/A
Muscle structure	N/A	MRI	Cadaver measurement	N/A	N/A
Elements	82 011 elements 285 694 nodes	554 154 elements 620 899 nodes	N/A	N/A	130 429 elements 30 181 nodes
Validation	Based on brachial plexus injury reported clinically^219,220^	15 g front impact^87^ 4 g rear impact^143,221^ 7 g lateral impact^76^	Anatomical measurement^200,222,223^; 2.3 m/s rear impact^208^	Quasi-static Flx/Ext/LB/AR^73^	Quasi-static Flx/Ext/LB/AR^73,74,224^
Application	Kinematics in pathological conditions of brachial plexus injuries	Influence of neck active force on the head–neck dynamic response and whiplash injury (low-speed collision)	In John, 2019^216^, Purushothaman, 2021^225^: effect of variations in cervical spine morphology, sex and head inertia properties on segmental rotation in rear-impact whiplash loading/G_-x_ accelerative loading; in Yoganandan, 2020^226^: effects of different severities of disc degeneration on the range of motion of cervical spine based on the John, 2019^216^; in Choi, 2020, 2021^227,228^, John, 2020^229^, Purushothaman, 2020^230^: kinematics and tissue responses in the index and adjacent levels of the human cervical spine after ACDF, and ADR with different types; influence of sagittal alignment of the cervical spine on the development of radiological adjacent segment pathology after central corpectomy. In Varghese, 2022^231^: a normalization technique was developed for scaling MRI data into FE model	Influence of extragraft bone formation on operated motion segment after ACDF	Biomechanical strategy for hybrid surgical strategy in three-level cervical degenerative disc disease
First author/year	Bailly, 2020^49^	Herron, 2020^50^	Hua, 2020^232^	Li, 2020^233^	Subramani, 2020^234^
Type	FE	FE	FE	FE	FE
Software	Radioss	FEBio	OptiStruct	ABAQUS	LS-DYNA
Segments	C2–T1	C0–T1	C2–C7	C2–T1	C2–C7
Figure	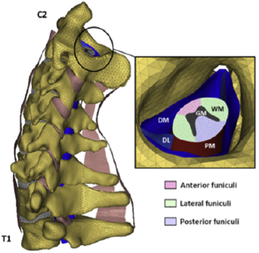	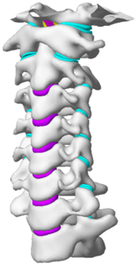	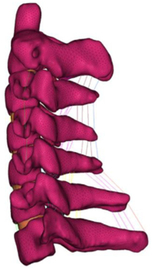	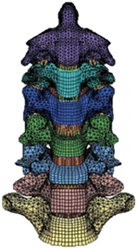	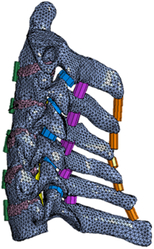
Geometry	CT	Previous FE model and visible human project	CT	CT	BodyParts3D
Head	N/A	Rigid	N/A	N/A	N/A
Vertebra	Rigid	Orthotropic elastic/neo-Hookean	Elastic	Elastic	Elastoplastic
Disc	Hyperelastic (annulus matrix/nucleus pulposus) Nonlinear spring (annulus fibrosis)	Hyperelastic (annulus matrix/nucleus pulposus) Fiber-exponential-power (annulus fibrosis)	Elastic	Elastic	Hyperelastic (annulus matrix/nucleus pulposus)
Facet joint	Frictionless contact	Sliding-tension-compression contact	Frictionless contact	Frictional contact	Contact
Ligament	Nonlinear shell	Nonlinear spring	Linear contact elements	Elastic	Linear beam
Spinal canal	Elastic (dura mater/pia matter/denticulate ligaments) Viscoelastic (white matter/grey matter)	N/A	N/A	N/A	N/A
Muscle	N/A	N/A	N/A	N/A	N/A
Muscle activation	N/A	N/A	N/A	N/A	N/A
Muscle structure	N/A	N/A	N/A	N/A	N/A
Elements	391 941 elements 90 523 nodes (spinal canal excluded)^62^	185 826/185 382/164 118 elements (3 models, respectively)	2 908 691 elements 783 503 nodes^235^	152 608 elements 41 797 nodes	N/A
Validation	FSU moment-rotation of 500 deg/s hyperextension^236^; In Beauséjour, 2020^62^: Quasi-static Flx-Ext^115,149^; IDP (Flx) in C3-C4, C5-C6^237^	Quasi-static Flx/Ext/LB/AR^14,73,104,153,209,238–240^; Facet Contact Force^238,241,242^; IDP in C2-C3, C6-C7^237,243,244^; Disc measurements^245^	Quasi-static Flx/Ext/LB/AR^18,73^	Quasi-static Flx/Ext/LB/AR^115,129,130,246^	Anatomical measurement^247,248^; Quasi-static Flx/Ext/LB/AR^73^
Application	In Bailly, 2020^49^, Lévy, 2020^249^, Beauséjour, 2020^62^: effect of spinal cord compression types, disc bulging and ligamentum flavum hypertrophy on degenerative cervical myelopathy; effect of injured posterior ligamentous complex and intervertebral disc on post-traumatic cervical spine instability	Biomechanical behaviours of morphological differences between various FE models	In Hua, 2020^232,235^: kinematics and tissue responses of ASD after one-level or two-level ACDF (cage-plate, zero-profile), and ADR	In Li, 2020, 2021^233,250^: impact of ASD after single-level ACDF with zero-profile versus cage-plate construct	Fatigue damage prediction in the annulus of cervical spine intervertebral discs for neck pain
First author/year	Sun, 2020^251^	Wong, 2020^51^	Barker, 2021^252^	Cao, 2021^253^	Nasim, 2021^254^
Type	FE	FE	FE	FE	FE
Software	ABAQUS	ABAQUS	LS-DYNA	ANSYS	LS-DYNA
Segments	C2–C7	C2–C7	Head–neck	C1–C7	Head–neck
Figure	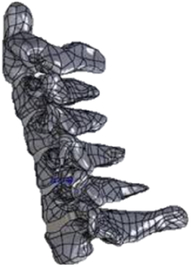	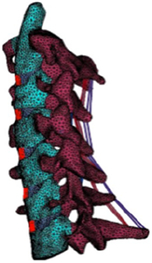	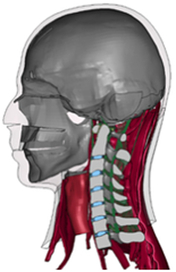	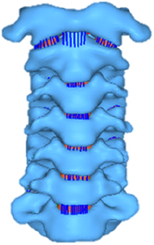	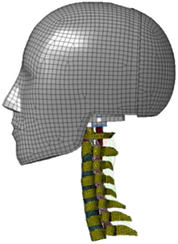
Geometry	CT	CT	CT/MRI	CT	Morphometric
Head	N/A	N/A	Elastoplastic	N/A	Rigid
Vertebra	Elastic	Elastic	Elastoplastic	Elastic/Elastoplastic	Elastic
Disc	Elastic	Elastic	Viscoelastic (annulus matrix) Orthotropic nonlinear elastic (annulus fibrosis) Elastic fluid (nucleus pulposus)	Elastic (annulus matrix/fibrosis) Hyperelastic (nucleus pulposus)	Elastic (annulus fibrosis) Viscoelastic (nucleus pulposus)
Facet joint	Frictionless contact	Frictional contact	Contact	Contact	Contact
Ligament	Linear truss	Nonlinear truss	Nonlinear spring	Linear spring	Nonlinear spring
Spinal canal	N/A	N/A	N/A	N/A	Hyperelastic (spinal cord)
Muscle	N/A	N/A	3D solid elements (passive muscle) Hill-type 1D spring/cable (active muscle)	N/A	N/A
Muscle activation	N/A	N/A	Predefined	N/A	N/A
Muscle structure	N/A	N/A	MRI modelling	N/A	N/A
Elements	N/A	222 788 elements	253 397 elements	N/A	94 997 elements 26 908 nodes
Validation	Quasi-static Flx/Ext/LB/AR^73,210,255,256^	Quasi-static Flx/Ext/LB/AR^73,74,224^; IDPs in C2-C3, C6-7^257,258^; Facet contact force^107,259^	Ligamentous cervical spine for axial tension^166^, AR^169^, and 8 g frontal/rear impact^145^; Full neck PMHS for 5–10 g rear impact^260^; full neck volunteer for 2–15 g frontal impact^77,172,261,262^, and 4–7 g lateral impact^77,263^	Quasi-static Flx/Ext/LB/AR^73^	Neck force-time for compressive impacts^165,264,265^; Quasi-static Flx/Ext^115,132^
Application	Kinematics and tissue responses of noncontiguous ADR and noncontiguous ACDF in the treatment of noncontinuous cervical degenerative disc disease	Optimization of three-level cervical hybrid surgery to prevent ASD	Biofidelic kinematic and local tissue response of full head-neck in male HBM	Biomechanical performances between single-level triangular and quadrilateral profile anterior cervical plates	Implemented head-first compressive impacts toward the assessment of motorcycle neck protective equipment
First author/year	Ovsepyan, 2021^266^	Sun, 2021^267^	Wo, 2021^268^	Xiao, 2021^269^	Xie, 2021^270^
Type	FE	FE	FE	FE	FE
Software	ABAQUS	ANSYS	ANSYS	ABAQUS	ABAQUS
Segments	Head–neck	C2–C7	C2–C7	Head–neck	Head–neck
Figure	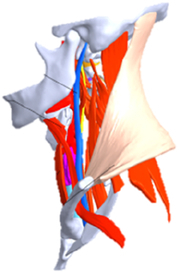	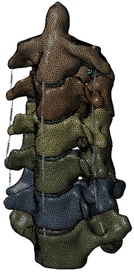	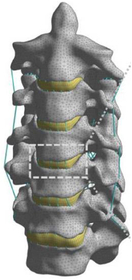	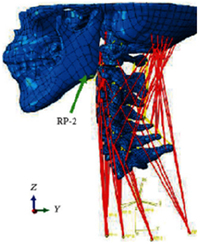	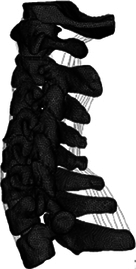
Geometry	CT	CT	CT	CT	CT
Head	Elastic	N/A	N/A	Elastic	Elastic
Vertebra	Elastic	Elastic	Elastic	Elastic	Elastic
Disc	Hyperplastic (annulus fibrosis/nucleus pulposus)	Elastic	Elastic	Elastic	Elastic
Facet joint	Contact	Frictional contact	Frictional contact	N/A	N/A
Ligament	N/A	Linear spring	Linear spring	Linear spring	Nonlinear spring
Spinal canal	N/A	N/A	N/A	N/A	N/A
Muscle	N/A	N/A	N/A	1D linear spring elements of passive muscles	N/A
Muscle activation	N/A	N/A	N/A	N/A	N/A
Muscle structure	N/A	N/A	N/A	N/A	N/A
Elements	Approximately 1 010 000 elements	N/A	226 402 elements 446 263 nodes	86 710 elements 296 644 nodes	549 717 elements 184 802 nodes
Validation	C3–C5 for compression^61,271^	Quasi-static Flx/Ext/LB/AR^73,158,210^	Quasi-static Flx/Ext/LB/AR^73,74,150,151,153,272–277^	Quasi-static Flx/Ext/LB/AR^73,115^	Quasi-static Flx/Ext/LB/AR^103,272,273,278–280^
Application	Functional and dynamic anatomy of nonlinearity of soft tissues as well as local anisotropy in the human head-neck	A lattice topology optimization of cervical interbody fusion and FE comparison with bio-absorbable cage and regular cage	Kinematics and tissue responses of cervical ADR using cervical subtotal discectomy prosthesis, and implanted it in non-human primates as an in-vivo experiment	Clinical effect and tissue responses of a traction exercise neck brace on cervical spondylopathy radiculopathy	Kinematics of the craniovertebral junction after odontoidectomy with anterior C1 arch preservation
First author/year	Nishida, 2021^52^	Sun, 2022^53^	Liang *et al*.^38^	Li, 2023^281^	
Type	FE	FE	FE	FE	
Software	ABAQUS	ABAQUS	LS-DYNA	LS-DYNA	
Segments	C2–C7	C2–T1	Head–neck	Head–neck	
Figure	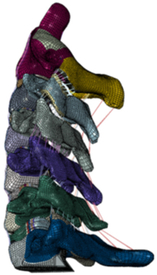	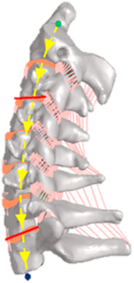	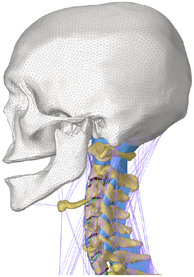	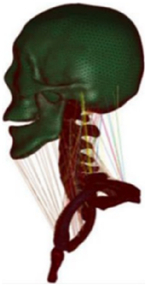	
Geometry	CT	CT	CT/MRI	CT	
Head	N/A	N/A	Rigid	Elastoplastic	
Vertebra	Elastic	Elastic/Neo-Hookean	Elastoplastic	Elastoplastic	
Disc	Hypoelastic (annulus matrix/annulus fibrosis/nucleus pulposus)	Hypoelastic (annulus matrix/annulus fibrosis/nucleus pulposus)	Hyperelastic (annulus matrix) Elastic fluid (nucleus pulposus) Orthotropic nonlinear elastic (annulus fibrosis)	Elastic (annulus matrix) Plasticity (annulus fibrosis) Viscoelastic (nucleus pulposus)	
Facet joint	Sliding contact	Soft contact	Contact	Bound contact	
Ligament	Nonlinear spring	Nonlinear spring	Nonlinear membrane	Nonlinear spring	
Spinal canal	N/A	N/A	Elastic (dura mater/pia matter/denticulate ligaments) Viscoelastic combined with hyperelastic (white matter/grey matter)	N/A	
Muscle	N/A	N/A	1D beam elements of Hill muscles (active muscle)	1D spring elements of Hill muscles	
Muscle activation	N/A	N/A	Coupled with neuromuscular feedback^58^	Predefined	
Muscle structure	N/A	N/A	Cadaver measurement^199^	Cadaver measurement	
Elements	213 165 elements 173 215 nodes	N/A	1 073 668 elements 246 523 nodes	296 487 elements	
Validation	Quasi-static Flx/Ext/LB/AR^224^; Facet joint forces and IDPs in C2-C6^224,237,241,243^	Cervical lordosis measurement^282^; maximum heights of intervertebral discs measurement^283^; Ligament lengths measurement^284^; Quasi-static Flx/Ext/LB/AR^73,115,129,130,210^; Facet joint forces in C2-C7^50,241,242^; IDPs in C2-C6^210,243,285^	Anatomy measurements (cervical lordosis, vertebral height, vertebral depth, vertebral width, and spinous process length)^200^; Compressive impact of spinal cord^286,287^; Ligaments tension of UCS and LCS^202,204,288^; FSU compression test^271^; Quasi-static Flx/Ext/LB/AR^73,115,132,149^; Kinematic and dynamic response of 4 g rear impact for whole head-neck (PHMS)^289^; Kinematic and dynamic response of 4 g rear impact for whole head-neck (volunteer experiment)^221^; Epidemiologic data reviewed	Head and neck axial impact experiment^290^; kinematic and dynamic response of 10 g and 15 g front impact for whole head-neck^72^	
Application	In Nishida, 2021, 2022^52,291^: influences of soft tissue injury in normal cervical spine and laminoplasty scenarios	Continuous biomechanical effects of follower load on the ROM, tissue responses of cervical spine	Protective effect of muscle activation caused by natural neural reflex on neck in impact loading by decreasing tissue responses and injury position to reduce trauma; quantitatively biomechanical response analysis of posterior musculature reconstruction in cervical single-door laminoplasty^292^	Effect of muscle activation on dynamic responses of neck of pilot during emergency ejection	

ACDF, anterior cervical discectomy and fusion; ADR, artificial disc replacement; ASD, adjacent segment degeneration; CT, computed tomography; FE, finite element; FSU, functional spinal unit; GHBMC, Global Human Body Models Consortium; HBM, human body model; IDP, intradiscal pressure; MIS, minimally invasive surgery; KTH, Kungliga Tekniska Högskolan; N/A, not application; OPLL, ossification of posterior longitudinal ligament; RMSE, root mean square error; rRMSE, relative root mean square error; SCI, spinal cord injury; THUMS, Total Human Model for Safety.

**Table 2:** Multipage tables of human head–neck multibody models within retrieved studies in the last two decades.

First author/year	Chancey, 2003^293^	Van Der Horst, 2004^46^	Galbusera, 2006^294^	Ahn, 2007^295^	van Lopik, 2007^296^
Type	MB	MB	MB	MB	MB
Software	LS-DYNA	MADYMO	VisualNastran	VisualNastran	VisualNastran
Segments	Head–T1	Head–T1	C2–C7	C2–T1	Head–T1
Figure	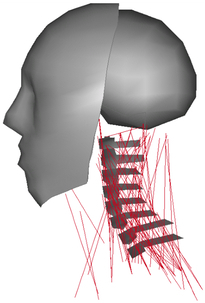	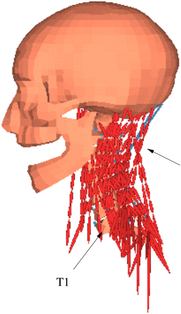	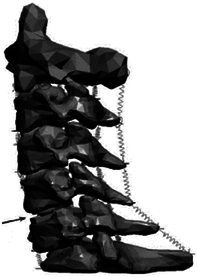	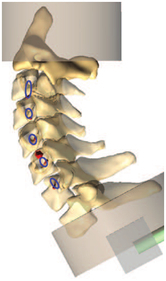	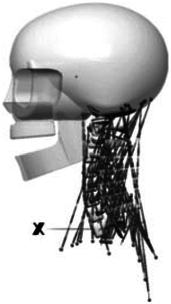
Geometry	CT/MRI	Visible human subject	CT	CT	CAD modelling
Head	Viscoelastic rigid face	Rigid	N/A	N/A	Rigid
Vertebra	Rigid	Rigid	Rigid	Rigid	Rigid
Disc	Couple superior vertebra to next inferior vertebra	6-DOF springs and dampers	N/A	Linear visoelastic	Viscoelastic bushing elements (Flx/Ext); linear (LB/AR)
Facet joint	N/A	Contact	Contact	Contact	Frictionless contact
Ligament	Nonlinear spring	Nonlinear spring	Linear spring	Nonlinear spring	Viscoelastic spring
Muscle	Nonlinear passive and active elastic components (optimization minimizing muscle fatigue)	Active muscle: Hill types	N/A	N/A	Active muscle: Hill types
Elements	N/A	N/A	N/A	N/A	N/A
Validation	In Oi, 2004^297^: Muscle moment arms comparison^154,298–300^	ROM of intervertebral units^74,165,301^; dynamic response to frontal/lateral Impact^172,302^	ROM of intervertebral units^303–305^	ROM of intervertebral units^73^ Axis-disc angle^306^	ROM of intervertebral units; Coupling characteristics; Ligaments’ response^74,81,165,274^
Application	Improved estimation of human neck tensile tolerance according to anthropometrically correct muscles and optimized physiologic initial conditions	Human had neck response in frontal, lateral and rear end impact loading	Constructed the flexion-extension motion of cervical spine after ADR surgery	Visualized realistically the kinematics and kinetics of the cadaver cervical spine	Evaluated realistic effect of curved musculature representation of the change in muscle length during the head-neck main and coupled motions
First author/year	de Jongh, 2008^48^	Huber, 2013^307^	Khurelbaatar, 2015^308^	Happee, 2017^22^	Cazzola, 2017^309^
Type	MB	MB	MB	MB	MB
Software	LifeMOD	Adams	RECURDYNTM	MATLAB	Opensim
Segments	Whole body model	C1–C7	Head–thoraco–Pelvis	Head–T1	Whole body model
Figure	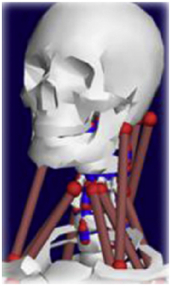	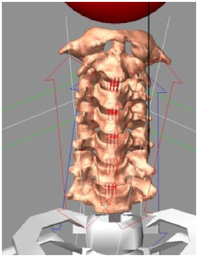	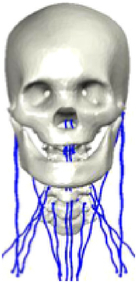	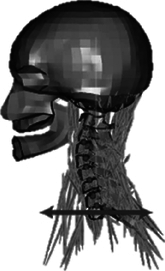	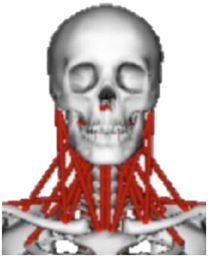
Geometry	Standard GeBod anthropometric database	White light scanner	CT	MRI and CT	3D digitizer
Head	Rigid	Rigid	Rigid	Rigid	Rigid
Vertebra	Rigid	Rigid	Rigid	Rigid	Rigid
Disc	6-DOF bushing elements	Bushing force	6-DOF bushing elements	6-DOF bushing elements	6-DOF elements
Facet joint	Contact	Contact	Plane-sphere contact	Contact	Contact
Ligament	Nonlinear spring	Force vector	Tensile-only spring	N/A	N/A
Muscle	Active muscle: Hill types	EMG	Active muscle: Hill types	Active muscle: Hill types	Active muscle: Hill types
Elements	N/A	N/A	N/A	N/A	N/A
Validation	Intradiscal pressure^310^; ROM of intervertebral units^311^	In house: internal load (EMG/optical data) compare to the external load (force plate)	ROM of intervertebral units^73,115,129^; ligament tensile forces^311^; Force of facet joint^18,312^	Head–neck dynamic responses during bandwidth of pseudorandom^313^	Scapula-clavicular joint and moment arms^314,315^; ROM of intervertebral units^272,273,305,316–318^; neck muscles activation during functional movements (EMG)
Application	Estimated time-varying contact stress and slip velocity distributions at the interface of the cervical disc implant and provided insight into the in vivo biomechanical performance of the implant’s wear rate	Predicted motion, moments on the cervical spine, and calculate loads on the discs as well as co-activation in the muscles that maintain balance of the head-neck	Predicted joint forces and moments applied to vertebral and facet joints and the forces that act on ligaments and muscles in cervical spine	Modulation of head-in-space and head-on-trunk stabilization strategy with the frequency content of trunk perturbations and the presence of visual feedback by vestibulocollic reflex, cervicocollic reflex, and neck muscle co-contraction	Investigated kinematics, joint moments and neuromuscular activations during rugby scrummaging and neck functional movements to explore cervical spine injury mechanisms
First author/year	Diao, 2017^319^	Mortensen, 2018^320^	Kuo, 2019^47^	Zheng, 2021^58^	Arshad, 2022^321^
Type	MB	MB	MB	MB	MB
Software	AnyBody	Opensim	Opensim	Opensim	AnyBody
Segments	Head–neck model	Head–T1	Head–T1	Head–T1	Head–neck model
Figure	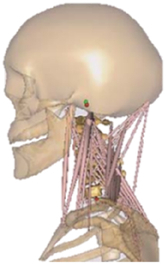	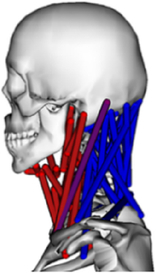	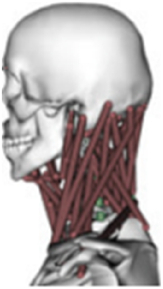	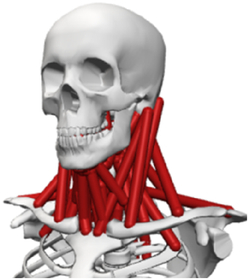	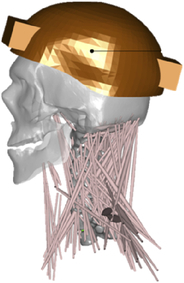
Geometry	AnyBody modelling system repository	Hyoid muscles improved base on previous MB model	MRI and CT	Neuromuscular reflex improved base on previous MB model	Visible human project
Head	Rigid	Rigid	Rigid	Rigid	Rigid
Vertebra	Rigid	Rigid	Rigid	Rigid	Rigid
Disc	6-DOF elements	6-DOF bushing elements	6-DOF bushing elements	6-DOF bushing elements	Spherical joints
Facet joint	Plane-sphere contact	Contact	Contact	Contact	N/A
Ligament	Linear spring	Rotational stiffness and damping	Nonlinear spring	Rotational stiffness and damping	Nonlinear spring
Muscle	Active muscle: Hill types	Active muscle: Hill types	Active muscle: Hill types	Active muscle: Hill types	Active muscle: Hill types
Elements	N/A	N/A	N/A	N/A	N/A
Validation	RMSE / $R^{2}$ for sensitivity analysis; maximum compressive forces of disc^322^; facet joint force^18,210^; ligaments’ force^308^; ICR A–P translation^323^	Previously published validation by both Vasavada and Cazzola^309,324^	Response of cervical spine ligaments during head impacts	Posture control experiment (EMG); Cervical joint angles in stable status [X-ray results]; 2/3/8/15 g frontal impact^325^	Correction factor of IDP^326–328^; axial and shear loads^329^; qualitatively compared 34 group muscles^330–332^
Application	Simulated the function of musculature and consider joint internal motion in the head–neck	Capability of hyoid muscles to stabilize the upper cervical spine and provide increased moment generation in head–neck	Stiffen effect of passive ligaments on providing greater deceleration angular impulses than active muscles at high lengthening rates in American football head impacts	Influences of realistic neural reflex control loops of the vestibular system and the muscle-tendon proprioceptors on in-vivo head-neck behaviors and related injury risk during impact loading	Quantifying the differences in disc loads, motion, and muscle force/activities through changing the vary in segmental mass, disc stiffness, and muscle strength

3D, three dimensional; ADR, artificial disc replacement; A–P, anterior–posterior; CT, computed tomography; ICR, Instantaneous centers of rotation; MB, multibody; *R* ^2^, correlation coefficients.

The first step in head and neck simulation tasks is to generate models for specific subjects, with two primary methods for creating personalized models. The first method involves scaling a generic model, while the second involves creating models based on the segmentation of medical images^25,26^. The primary task in medical image-based processing is to collect medical images (such as MRI and CT scans), while bi-planar X-rays and three-dimensional ultrasound measurements can be utilized to identify specific anatomical landmarks for personalized models. Statistical shape modeling and morphing techniques aid in rapidly and accurately reconstructing bones^27^. Creating models from medical images is very time-consuming and requires extensive expertise. Therefore, the importance of rapid, personalized modeling methods has been increasingly emphasized in recent years^28,29^.

Conventional personalized scaling involves resizing the generic skeletal geometry according to individual dimensions. However, the greater the discrepancy between an individual’s anthropometry and the generic model, the less likely the scaled model will accurately represent the anatomy^30^. Modeling based on weight and segment lengths allows for scaling generic models to specific individuals. The conventional scaling method is linear scaling^31,32^, which is the standard method for model examples included in the AnyBody modeling system’s user model repository. Proportional scaling is based on an individual’s height and body proportion, selecting the closest model for scaling to the individual^33^. Linear scaling of generic models, despite being time and cost-effective, probably results in significant errors due to not accounting for anatomical differences between individuals^29^. Marker-based scaling methods rely on the distance between virtual markers placed on the model and experimental marker positions provided by a photonic system^32,34^. This method uses a reference record to create a stick-figure model, with joint parameters directly calculated from optical markers placed on anatomical landmarks. The collected kinematic representation is then registered with a cadaver-based musculoskeletal dataset. During registration, a nonlinear transformation is created to scale (morph) the cadaver-based dataset to match the subject-specific joint parameters. Morphing utilizes radial basis functions (RBF) to interpolate data between markers. Thus, with an appropriate marker protocol, this method directly provides scaling of the musculoskeletal model. The RBF-based scaling method allows for scaling the entire model using a small number of anatomical landmarks, thereby reducing subjectivity and manual intervention in the scaling process^35^.

Computational biomechanics seeks to apply the principles of mechanics to living tissues. The results showed that linear materials were mainly used in FE models before 2005. Thereafter, the Kungliga Tekniska Högskolan (KTH) model^36^, the Total Human Model for Safety (THUMS) model^15^, and the Global Human Body Models Consortium (GHBMC) model^37^ were sequentially developed and taken into account the pronounced nonlinearity and anisotropy of human tissues. Compared to collision models in the same period, medical models materials were much simpler. Nonlinearity was mostly concentrated in intervertebral discs and spinal ligaments, which were defined as viscoelastic or hyperelastic characteristics. Although the current constitutive equation can well simulate the mechanical behavior of most spinal tissue, the contents within the spinal canal still need to be improved in terms of modelling and materials. Recently, a hyperelastic Ogden material with linear viscoelasticity (prony series) based on recent experimental studies of in-vivo nonhuman primates was used in Liang *et al.* ^38^. However, the mechanical behavior of the synchronized interaction between fluid (blood vessel, cerebospinal fluid) and structure (spinal cord) is currently neglected in head–neck computational models. More experimental studies on spinal cords are needed in the future for more accurate FE modelling.

Validation always plays an important role in the field of computational biomechanics; this field has utilized validation in an attempt to build credibility for models of complex biological systems. As shown in Table 1, the developed FE or MB models used different types of validation methods, which subsequently increased and extended their applicability according to different research needs. Comparisons of segmental ROM between model predictions and experimental measurements were mostly performed under quasi-static or dynamic working conditions, and examples include Ng *et al.* ^39^, Zhang *et al.* ^40^, del Palomar *et al.* ^41^, Tchako and Sadegh^42^, etc. In addition, FE or MB models for collision applications validated velocity and acceleration parameters to verify complex dynamics responses^36,43–47^. Recently, studies validated the use of internal parameters to compare the results of in-vitro measurements, such as intradiscal pressure and facet joint force^48–53^. Anatomical measurement for validation has also been performed in recent years because of the inevitable modifications in the process of geometric reconstruction^38^. Moreover, recent numerical models have recognized that the involvement of active muscles under neuromuscular feedback plays an important role in the understanding of fundamental principles of human head–neck motion. Efforts have been made to include more complicated control strategies, from predefined active curves^54,55^ or simple PID control^56,57^ to complex physiological neural feedback control strategies^38,58^, in these models. These models in studies were mostly developed to investigate the precise mechanism of cervical spine injuries during sports and traffic collisions.

In addition, the application of computational HBMs revolved around four specific subjects in previous studies: physiopathology, treatment evaluation, collision condition, and sports injury. Based on previously established and validated head–neck computational models, most of the studies investigated cases in depth. Examples included collision, sports injury, a specific pathological model, and instrument assessment. Head–neck computational models in the first decade mostly focused on collision studies, while models applied to large numbers of medical environments in the second decade. The advent of computational models in clinical use stemmed from the integration of biomechanical research and medical needs: clinical researchers gradually realized that biomechanical simulation analysis could play an increasingly important role in cervical spine diseases and treatments^59,60^. HBMs assisted in understanding the biomechanical mechanisms underlying disease progression, aiding in early disease diagnosis and the formulation of treatment plans^19,61–63^. They also could be utilized to plan surgical procedures, simulate surgical operations, and evaluate their outcomes and risks^64–66^. Hence, it is thus not a surprise that biomechanical research on medical applications has increased at a rapid pace.

## Discussion

In the last two decades, there has been a yearly rise in the volume of new research in the field of biomechanics relating to the human head–neck. This review places the findings regarding biomechanical modelling, validation, and application of the head–neck part into the context. While conducting a new study is always important, researchers must familiarize themselves with and establish the efforts of their predecessors. To date, the most common head–neck HBMs are adopting FE and MB modelling methods. The former puts particular emphasis on the loading condition, stress responses, and strain responses of local human tissues, while the latter comprises rigid skeletons and one-dimensional muscle components that generally aim to analyze the whole dynamic responses of the head–neck. Due to theoretical differences, although the MB models cannot be used to monitor tissue stress/strain responses in general, they are more computationally efficient in the analysis of dynamic responses than FE models. Although different controllers have been implemented in recent FE head–neck models^22,56–58,198,333,334^, the larger computational demands inherent in FE models can still restrict their development in modelling methods and their biofidelity, such as their ability to incorporate complicated neural control strategies^56,57,198^. However, these features have been continuously improved in MB models. These respective features probably lead to various environments concerning simulation requirements^22,58^.

**Figure 3:**
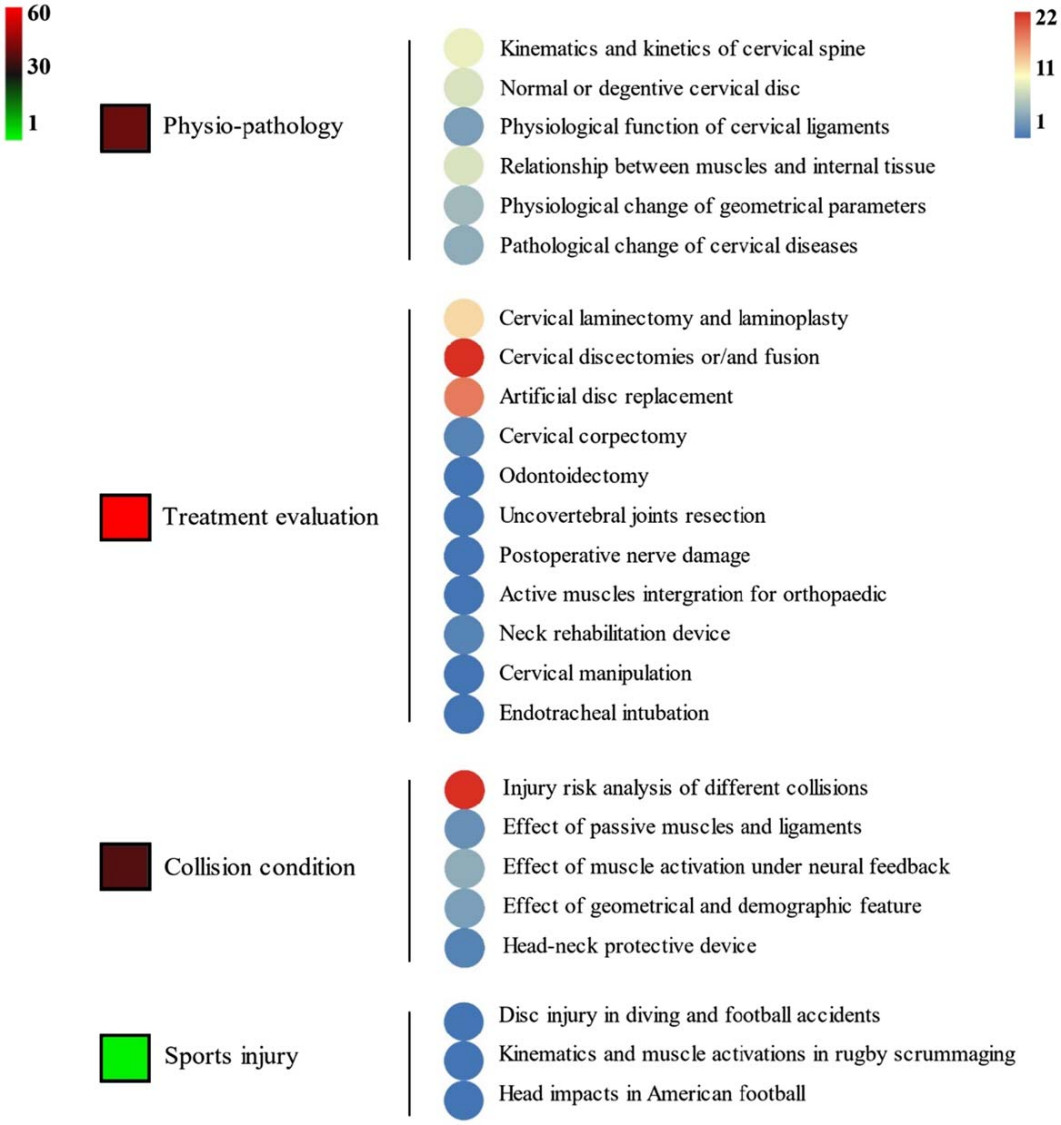
Heat map of application in head-neck computational models within four specific subjects.

**Figure 4:**
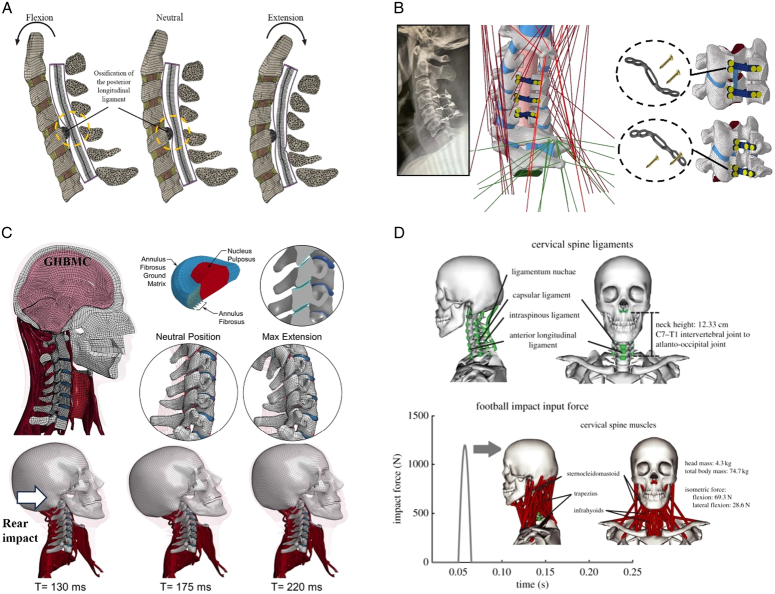
(A) Pathological cervical spine FE model for the spinal cord compression of ossification of the posterior longitudinal ligament^190^; (B) Modified muscle-preserving techniques of single-door laminoplasty in cervical spine surgery through head-neck FE model simulation^292^; (C) 50th percentile male of GHBMC head-neck FE models under rear impact kinematic sequence that simulated whiplash injury^354^; (D) Anatomical loads and mechanisms of cervical spine injuries associated with rugby in head-neck multibody model. FE, finite element; GHBMC, Global Human Body Models Consortium.

During the last two decades, the combination of basic and clinical orthopedic knowledge with biological modelling technology has shown a multidisciplinary trend in modern orthopedic research^335^. Many clinical problems of the cervical spine, such as mechanical neck pain through musculoskeletal disorders, indeed need a biomechanical model that can reveal the essential connection between internal human tissue and external intervention. Hence, the application of physiopathology and treatment evaluation gradually exceeded the observed impact scenarios during the last decade and became the most attractive feature of head–neck biomechanical modelling. In addition, the development of computer science and the use of new imaging technology increased the efficiency of simulation and modelling^336,337^. As shown in Figure 3 and Figure 4, the studies included in this review approximately revolved around four specific subjects: physiopathology, treatment evaluation, collision condition, and sports injury. Medical scenarios should still be a focus of future research efforts, given the application fields of computational head–neck models.

### Physiopathology

At present, inherent differences in human anatomical structures and material properties among individuals and alterations in these parameters due to age, sex, and degeneration can limit the widespread applicability of the reported models. For example, most FE models are generally based on one specific or one idealized subject (exactly symmetrical) with unique mechanical and geometrical characteristics, and some studies have explored the influence of geometric factors in subjects on biomechanical responses, including kinematic change, tissue stress and strain in the head–neck region^50,55,163,175,180,196^. If a model aims to predict the behavior of an average subject, it should incorporate average anatomical properties and be fully validated for biomechanical responses to increasing predictive ability^338,339^. Although most biomechanical researchers are aware of the importance of validation, many detailed FE models of medical applications only validated quasi-static segmental ROM. Merely a small fraction of models additionally validate intradiscal pressure and force of facet joint^49–53^. These parameters provide insights into the internal loading conditions of the cervical spine. In addition, for a variety of conditions from quasi-static loading to dynamic loading, many detailed models lack multilayer model validation from anatomic verification to isolated tissues to subsegments and the whole head–neck model. Inadequate model verification and validation can substantially reduce the robustness of model simulation results in various loading environments. There are certain concerns associated with these environments, including challenging validation processes and the application of the model beyond its validation boundaries^338^. This implies differences in the definition of loading cases to investigate injuries; on the other hand, it provides differences in terms of their level of accuracy for injury prediction.

Pathological models were established to evaluate underlying variables based on validated mathematical models. The FE model of spinal cord injury (central cord syndrome), which is induced by degenerative cervical myelopathy or ossification of the posterior longitudinal ligament (OPLL), is mostly modelled in the cervical segment, as shown in Figure 4. Imaging scans ignore the dynamic pressure-induced changes in degenerative structures in the spinal canal, especially for cervical spines with high mobility^340^. Finding a potentially compressive source (disc bulging, ligamentum flavum hypertrophy, etc.) is often an important basis for the early diagnosis of central cord syndrome. FE predictions undoubtedly have higher sensitivity in terms of the respective mechanical behavior of the detailed pathological or clinical type^19,188,189,249^, which can fully display dynamic changes in the spinal canal under head–neck motion conditions. Thus, the use of biomechanical simulation models can undoubtedly be an essential tool to assess the best clinical option^341^.

The biomechanical characteristics of the FSU are fundamental for understanding the cervical spine and its various pathologies. Studies on basic mechanisms that support load and enable head–neck movement help to explain clinical problems of musculoskeletal disorders, such as those related to the relationship between internal loads and external muscle forces^42,293,308,320^, FSUs under a superimposed compression force (follower load)^53^, cervical disc stiffness^123,226,321^, facet joint orientation^175,196^, etc.

### Treatment evaluation

Based on the established pathological models, corresponding treatments were analyzed and evaluated biomechanically. Intervertebral fusion and artificial disc replacement are most widely studied in cervical surgery. Discectomies and facetectomy produce iatrogenic injuries that include altered structural responses and redistributed load in the FSUs^39,123^. To avoid segmental instability, clinicians implanted an interbody cage or artificial disc replacement into the lesion segment. Substantial research over the past 20 years has simulated the responses that occur after prosthesis or osteosynthesis implantation using both FE and MB models. Additional advantages of the FE models are that they allow predictions of the key parameters of mechanical stability, including the cervical Cobb angle, the pull-out force of cages, and stress responses around the zones of implanted screw-plate, cage, or prosthesis. Moreover, by setting different contact relationships between the cage–endplate interface or growth factors of bone creeping substitution, FE models can even predict different periods of intervertebral osseointegration^124^. Although existing biomechanical models cannot accurately simulate postoperative cage subsidence or prosthesis migration, the models can observe the stress and its distribution on the cage–endplate interface and cancellous bone under the bony endplate as the risk of subsidence and migration. These mechanical responses not only prevent future pathologies derived from an unsuitable positioning of the prostheses or its fixation but also serve as a basis for the design and optimization of dimensions, cavity volume, position, material, fixation methods, and structural characteristics of instrumented implants^65,267^.

Apart from anterior cervical spinal fixators, posterior fixators have also been widely studied, especially in the first decade of analysis in this review. Laminectomy and laminoplasty are representative surgeries for OPLL. These biomechanical studies are almost all from East Asia because OPLL is fairly common in Asian individuals and is defined as a rare disease in Western countries^342,343^. Sutures, titanium cables, suture anchors, allograft or autograft bone, synthetic spacers, and miniplates were used in laminoplasty, and current studies primarily compared the biomechanical benefits and complications of instrumented implants^19,63,93,133,135,189–191^, as shown in Figure 4B. Different from cervical fusion, laminectomy, and laminoplasty preserve the motor function of the intervertebral disc. However, muscle-derived axial neck pain, the most common complication, decreases postoperative ROM and has a negative effect on treatment outcomes^344^. Moreover, the active muscle force should be considered when evaluating muscle-derived axial pain, but there is a lack of biomechanical studies on the active muscle behavior of laminectomy or laminoplasty, which is surprising considering that these two surgeries are being performed clinically.

Muscle structure and function should be purposefully added to medical models, which would undoubtedly increase the accuracy of clinical treatment predication and evaluation. Howley *et al.* ^182^ modelled and integrated three-dimensional deformable muscles with contraction capabilities in an effort to pave the way for various orthopedic simulations. It is believed that biomechanical research for orthopedic and other medical environments will continue to increase. Minimally invasive cervical surgery is the future direction in clinical practice, and now its biomechanical evaluation is gradually being reported^66,161^. Furthermore, rehabilitation, anesthesia, and alternative medicine started to adopt biomechanical modelling in recent years to solve clinical issues, such as postoperative complications^189^, conservative strategies^194,195,269^, endotracheal intubation^197^, etc.

### Collision condition

Compared with the detailed FE models for medical applications, models for collision conditions were developed for a longer period and seem to be more comprehensive and realistic. Several whole HBM have also been developed^15,37,43,45,199^ (Fig. 4C). In the past decade, an increasing number of FE HBMs for impact injury analysis have consisted of active muscle modelling to reflect the effects of muscle activities on human body injuries, such as in emergency braking and wheel operation^101,345–348^. First, predefined activation-time curves were defined for muscle activation modelling^54,84,137^. However, this simplified method cannot represent the complexity of the neural reflex control loop. Recently, artificial controllers of muscle activation were also gradually added to FE models to include neural reflex control loops responding to external stimulation. Of note, Putra and colleagues implemented a PID-based control strategy in the ViVA female OpenHBM that was first established by Östh and colleagues, which included an angle-based and length-based active muscle controller^14,57,198^. A similar method was also implemented in the head–neck part of the GHBMC model^56^ and the THUMS model^349^. However, these simplified sensory feedback control modelling methods were found to be limited in their reactions to environmental perturbation^350^; moreover, the computational cost is high, especially when the modelling complexity of the neural control method is increased to be humanlike^351^. Few studies adopted physiologically realistic mathematical models of the neural reflex of the peripheral sensory system and vestibular feedback, implemented them into MB models, and applied them in impact and perturbation analyses; these analyses revealed influences of these proprioceptors on head–neck dynamic responses^22,58^. However, stress and strain distribution and values in human tissue for detailed injury risk analysis cannot be easily and accurately obtained using the MB models due to their theoretical limitations.

Recently, Liang *et al.* ^38^ introduced the combination of the FE and MB head–neck models, who analyzed the Von mises stress or strain responses of local tissues in a whiplash-loading environment by coupling a neuromuscular head–neck MB model and a corresponding FE head–neck model. The results show that muscle activation caused by the neural reflex protects the neck during impact loading by decreasing the strain level and transferring the possible injury to the lower spinal cord level to reduce injury severity. Thus, the FE-MB coupled method with natural neural control modelling is an interesting research field that can provide unique findings from human body biomechanical analysis. This approach combines the advantages of FE and MB methods and enables head-neck models to facilitate stress and strain prediction in local tissue under realistic human body motion, which should be further developed for more accurate biomechanical prediction.

In addition to advanced control strategies, tissue materials of pronounced nonlinearity and local anisotropy are more often applied in collision applications than in the medical environment due to the large deformation in collision requirements. However, more complicated constitutive equations or material modelling methods can increase the computational cost and instability of FE HBMs in the solving process. Therefore, the applicability and simplicity of the HBMs need to be balanced. The FE HBMs for medical applications focus more on anatomical details, while the models for impact injury analysis probably simplify or idealize some anatomical structures. The former often focuses more on subtle tissue deformation in local medical intervention, and the latter is usually used in loading environments with large tissue deformation. Importantly, biological systems are inherently complex and comprised of multiple interdependent variables with subtle relationships to each other, which makes it prohibitively difficult to take all potential variables into account in the computational model^352^. Sometimes, errors are even caused by the ‘garbage in garbage out’ phenomenon that is inherent to any computational model^353^. Through the lens of physics and mechanics, biological concepts may conflict with the clinician’s inherent understanding.

In summary, multiple causes make standardize collision FE HBMs. First of all, the design goal of the crash FE HBMs is to comprehensively evaluate the human body injury in the collision process, so the simulation results of the main body injury parts need to be verified in detail. Therefore, models for collision conditions have a broader application than specific medical FE models. Secondly, there is no unified protocol for the establishment of medical detailed FE HBMs, while a complete set of verification protocols has been established for collision models after years of development since it is due to the early start of vehicle collision simulation model, forming a systematic, engineering unified simulation analysis standard. Models for collision conditions have been proposed for widely involved in future virtual evaluation of automobile safety under the relevant regulations. Due to the lack of complete establishment and verification specifications, the application of FE models for medical use is often limited to specific cases and specific conditions.

### Sports injury

MB models are ideal for estimating system dynamics during sports impact events, providing a viable approach to test fundamental principles and investigate their injury mechanisms. It also allows simulations to be run at low computational cost. However, the head–neck MB model is still in its infancy for sports injury applications. Recently, some relevant research attention has been placed on American football^355–357^, as shown in Figure 4D. It is a full-contact sport that, on occasion, can result in serious cervical spine injuries. Similar to whiplash injury, the primary injury mechanisms of the cervical spine in sports injuries are buckling or hyperextension^358^. A generic full-body MB model (Musculoskeletal model for the Analysis of Spinal Injuries) was developed to investigate cervical spine loading through both inverse and forward simulations^309^. Then, the model was improved by recent studies investigating collisions in sports and motor vehicles^47,58,320^. Likewise, the development of head–neck FE models and MB models has facilitated the impact of kinetics and injury metrics of head-neck resulting from various sports, including ice hockey^359–361^ and boxing^362^ in recent years. Levy *et al.* ^359^ conducted laboratory experiments to determine head kinematics and injury metrics in hockey using FE models. The researchers investigated the head acceleration and rotational velocity of a helmeted head form subjected to various impact scenarios. Similarly, Michio Clark *et al.* ^360^ utilized FE models to investigate the distribution of brain strain in the cerebrum for laboratory impacts on ice hockey goaltender masks. The study highlights the need for improved helmet designs that consider not only linear acceleration but also rotational acceleration and brain tissue deformation. In boxing, Razaghi and Biglari^362^ conducted a comparative numerical model to simulate the ocular injury, which demonstrated the importance of using FE models to predict and analyze ocular injuries in boxing. Li *et al.* ^363^ also used a FE head model to simulate the boxing-type impact injury and found the lateral impact was more injurious to the brain than an anterior-posterior impact. Cournoyer and Hoshizaki^364^ analyzed the head dynamic response and brain tissue deformation for boxing punches with and without loss of consciousness using MB models. Their findings suggest that a loss of consciousness may be caused by a high level of head acceleration, which may lead to brain tissue deformation and neuromuscular responses.

Hence, over the past 10–15 years, there has been a large increase in the occurrence of sports-related spine injuries that result in severe trauma in the spinal cord, interverbal disc, articular capsule, etc.^365–368^. The design and optimization of subject-specific protective devices are expected to improve, especially in biomechanical models, which monitor head–neck kinematics and consequently reduce the risk of injury to the cervical spine in sports. For example, de Grau *et al.* ^361^ further explored the protective capacity of ice hockey helmets, who used FE models to investigate helmet performance at different levels of striking compliance. Their research found that helmets that offer higher levels of impact protection can better reduce head injury risk in ice hockey.

## Conclusion

This review systematically summarized existing head and neck models and discussed the novel findings regarding their modelling approach, structural characteristics, validation, and application. These methodologies of biomechanical models primarily focus on application-centred differences. Firstly, medical simulations have become the most researched content in head-neck biomechanical modelling over the last decade, while models for medical scenarios start later and develop slowly, which is probably due to limitations in the medical background. Tissue materials of pronounced nonlinearity have been less utilized in medical FE models when compared with models for collision, which is typically used in loading environments with large tissue deformation. Secondly, several models lack multiple-layer validation, such as anatomical verification, isolated tissues to subsegments, and the whole head–neck model, in a variety of conditions ranging from quasi-static loading to dynamic loading. More comprehensive validations that combine characteristics of interdisciplinarity will strengthen the utility of models and lead to multidirectional scenarios. Thirdly, advanced neuromuscular reflex control is being included in biomechanical head–neck models for collision applications, while this important control strategy has yet to be added to the medical models. It is suggested that the effect of active muscle control would enhance the understanding of treatment evaluation for medical head–neck models, and validate the models using real-world data. Finally, standardization in integrated medicine and engineering can greatly enhance the development and application of modeling processes in this field. By establishing standardized practices, guidelines, and frameworks, the integration of medicine and engineering can become more efficient, reliable, and effective.

## Ethical approval

Not applicable.

## Consent

Not applicable.

## Source of funding

This work was supported by the National Natural Science Foundation of China (Grant No. 52275286), Hunan Outstanding Youth Fund (Grant No. 2023JJ10010), Key Research and Development Program of Hunan Province (Grant No. 2022SK2105), Open Project of Xiangjiang Laboratory (Grant No. 23XJ03015), Shenzhen Science and Technology Program (Grant No. JCYJ20230807122004009), and the Sanming Project of Medicine in Shenzhen (Grant No. SZZYSM202311006).

## Author contribution

Z.L.: methodology, writing-original draft, and visualization; K.W.: reviewing and modification; T.T.: investigation and resources; F.M.: conceptualization, supervision, project administration, and funding acquisition.

## Conflicts of interest disclosure

The authors declare no conflicts of interest.

## Research registration unique identifying number (UIN)

Not applicable.

## Guarantor

Fuhao Mo.

## Data availability statement

All data generated or analyzed during this study are included in this article.

## Provenance and peer review

No invited.
